# Cardiac Arrest and Successful Extracorporeal Cardiopulmonary Resuscitation as a Result of a Refeeding Syndrome in a Young Female with Anorexia Nervosa

**DOI:** 10.1155/2020/8217583

**Published:** 2020-07-28

**Authors:** Daniela Waddell, Felix Meincke, Samer Hakmi, Hendrick van der Schalk, Niklas Schenker, Jonas Hahn, Anna Moschner, Mintje Bohné, Da-Un Chung, Stephan Willems, Dietmar Kivelitz, Edda Bahlmann

**Affiliations:** ^1^Department of Cardiology, Asklepios Clinic St. Georg, Hamburg, Germany; ^2^Department of Radiology, Asklepios Clinic St. Georg, Hamburg, Germany

## Abstract

Anorexia nervosa is a potentially life-threatening eating disorder, characterized by an abnormally low body weight. This case report illustrates a 22-year old female with cardiac arrest due to a refeeding syndrome in a patient with anorexia nervosa. It features the successful use of extracorporeal cardiopulmonary resuscitation in a case of severe left ventricular dysfunction resulting in a favorable outcome. *Conclusion*. We present the first case of a cardiac arrest due to a refeeding syndrome in anorexia nervosa featuring the successful use of an extracorporeal cardiopulmonary resuscitation approach as a bridge to full recovery.

## 1. Introduction

In female patients hospitalized due to eating disorders, nearly half of the patients were categorized as having AN [1]. AN is a psychiatric disease with a high mortality rate, 85% occurring in young women [2]. Reported mortality rates for AN range from 1.36% to 20% [3]. Death is commonly resulting from sudden cardiac death in one-third of all deaths associated with AN or suicide [4, 5]. However, sudden cardiac death in refeeding syndrome is not that frequent in AN [6–9]. In a case review, 3 of 48 adolescent females admitted with AN developed life-threatening complications associated with refeeding in AN [7]. Of these, one presented with a severe cardiac complication, an asystolic cardiac arrest [7]. Typically, but not in all cases of sudden death in AN with refeeding [9], these clinical findings are associated with hypophosphatemia [6]. This is the first report documenting cardiac arrest and successful extracorporeal cardiopulmonary resuscitation as a result of a refeeding syndrome in a young female with AN.

## 2. Case Presentation

**Figure 1:**
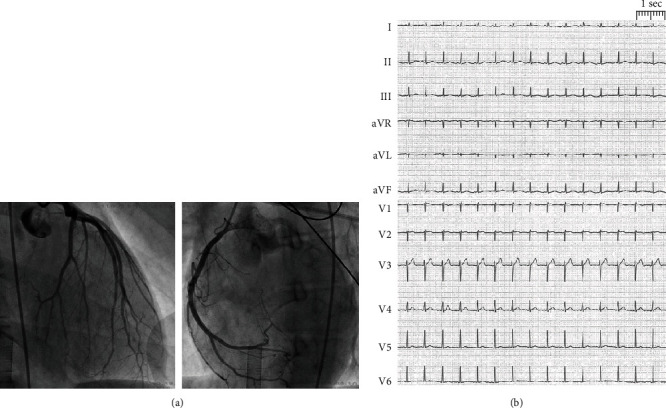
12-lead electrocardiogram shows sinus tachycardia (heart rate 98 beats/min), and prolongation of the corrected QT interval was detected (660 ms).

A 22-year-old female (height 1.72 cm, weight 40 kg, and body mass index 13.5 kg/m^2^) was admitted to our emergency room while receiving cardiopulmonary resuscitation for 45 minutes. Rhythm upon arrival was ventricular fibrillation. Since return of spontaneous circulation could not be achieved by all medical measures provided, the patient was immediately transferred to the catheter laboratory. Extracorporeal cardiopulmonary resuscitation via venoarterial extracorporeal membrane oxygenation (va-ECMO) was established. Due to severe left ventricular (LV) dysfunction, an Impella® pump was added to ensure sufficient LV output. Subsequently, percutaneous coronary angiogram was performed and showed no coronary disease (Figure 1(a)). The patient's history was noticeable for anorexia nervosa (AN) with several hospitalizations for renutrition, at the last several months prior to emergency admission. During the last few days prior to sudden cardiac death and after excessive caloric restriction, our patient began to increase her caloric intake to improve her nutritional state to avoid complications at an upcoming doctor's appointment. Prior to admission, our patient was an outpatient; did not receive medical refeeding or preventive treatment with supplementation of vitamins, phosphorus, or micronutrients; and did not had a history of binge episodes.

**Figure 2:**
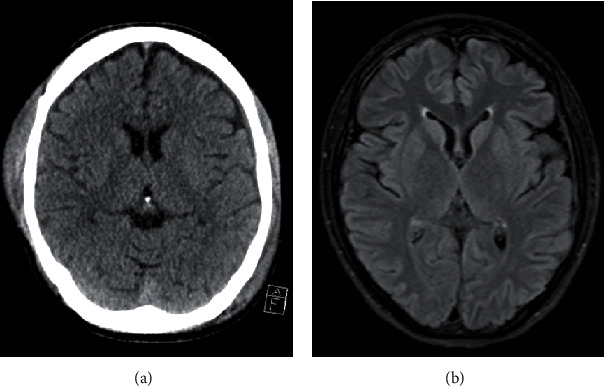
Cranial CT scan (a) and cranial MRI scan (b) revealed evidence of a small right-sided subdural and intracerebral hematoma.

**Figure 3:**
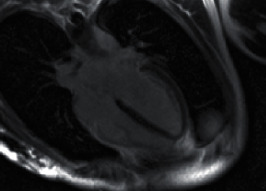
Cardiac MRI demonstrates a 4-chamber late enhancement view without pathological findings.

Initial blood analysis showed a severe electrolyte disorder with potassium depletion (1.38 mmol), hypocalcemia (ionized calcium 1.75 mmol/l), hyponatremia (117 mmol/l), hypochloremia (54 mmol/l), hypophosphatemia (0.28 mmol/l), and hypoproteinemia (total protein 30.2 g/l; albumin 28.9 g/l). In addition, laboratory data indicated a high level of lactate (18 mmol/l) combined with a normal pH of 7.38. Initial sugar levels were elevated (blood glucose 430 mmol/l). The first performed echocardiogram confirmed severe LV dysfunction. In the electrocardiogram, a sinus tachycardia and prolongation of the corrected QT interval were detected (660 ms) (Figure 1(b)). Electrolytes were substituted. Transient anisocoria and simultaneous severe coagulopathy under va-ECMO therapy led to a cranial computed tomography (CT), showing small bilateral subdural hematoma (Figure 2(a)). Most likely, the patient had aspirated during resuscitation; therefore, calculated antibiotic therapy with ampicillin/sulbactam was given. After initiating inodilatative support with levosimendan, cardiac output significantly improved within the next 24 hours. The Impella® pump and va-ECMO were subsequently removed on day 3. After the patient's respiratory situation had stabilized, extubation took place on day 5. Neurological assessment 10 days after admittance showed a mild difference in reflexes such as vertical saccade. Cranial magnetic resonance imaging (MRI), performed on day 16, revealed unchanged small bilateral subdural hematoma (Figure 2(b)). Subsequent cardiac MRI performed after 2 weeks showed a recovered LV ejection fraction and no signs of myocarditis (Figure 3). The patient was dismissed after 3 weeks of hospitalization without neurological deficit to a psychiatric care station for further treatment of AN.

## 3. Discussion

Silent LV dysfunction in patients with AN in the starvation phase is common [10], and its identification is of importance to prevent heart failure, a complication that often develops during clinical refeeding [6]. Causes of heart failure in AN patients with severe malnutrition often include deficiencies of magnesium, phosphorus, thiamine, and selenium [11–13]. It is suggested by de Simone et al. in 1994 that an abrupt increase in preload developing during refeeding might precipitate heart failure [10]. There were not enough elements for a chronic LV dysfunction preceding cardiac arrest in our patient. Rather, a massive LV dysfunction without an associated chronic LV dysfunction decompensated by a refeeding syndrome is likely. This hypothesis is supported by the imagery data and the rapid return “ad integrum” of the LV function after resuscitation. LV dysfunction together with the electrolyte abnormalities that can occur as a result of starvation and malnutrition caused by AN [14, 15] or during refeeding [6] (that is, hypomagnesaemia, hypokalemia, and hypophosphatemia, and hypocalcemia), severely present in our patient, can lead to sudden cardiac death in these patients [7, 10]. However, a more recent study with a more aggressive approach to nutritional rehabilitation to avoid ineffective refeeding in AN showed no evidence for developing moderate or severe hypophosphatemia [16]. Thus, a multivisceral failure associated with an increasing undernutrition, besides a refeeding syndrome hypothesis, might also be considered as a cause of cardiac failure in our patient.

Prolongation of the corrected QT interval due to the severe electrolyte disorder, present in our patient, is a known predictor of serious cardiac arrhythmias in AN [17]. Cardiotoxicity and arrhythmogenesis in these patients might also be related to elevated sympathetic activity [18]. Sudden cardiac death due to catecholamine cardiomyopathy is described in one case of a 22-year-old female [19]. Similar to the potentially lethal phenomenon of Takotsubo cardiomyopathy [20, 21], cardiac abnormalities in patients with AN are in fact reversible conditions after weight recovery [4, 22].

As usually described, refeeding syndrome in our patient also occurred within four days of starting to refeed [6–8]. Gradually increasing the caloric intake while carefully monitoring patients is therefore recommended for treating AN patients [23].

During fasting, the body switches its main fuel source from carbohydrates to fat tissue fatty acids and amino acids as the main energy sources. As result of a disturbed glucose metabolism and insulin secretion in refeeding, electrolyte disturbance typically takes the form of a hypokalemic, hypochloremic, and metabolic alkalosis [24]. The initial presentation with a pH-neutral severe lactatemia thereby likely is interpreted as a result of the prolonged hunger metabolism and also severe thiamin depletion. Dependent edema, often complicating refeeding and associated with lowered plasma proteins, could not be distinguished from treatment-dependent volume overload due to a severe cardiogenic shock in this case. Prolonged advanced life support including mechanical circulatory and temporary LV support devices in the setting of cardiac arrest unresponsive to resuscitation efforts and severely depressed LV function, as recommended in current guidelines [25], resulted in a favorable neurologic outcome in our patient.

In the presence of a reversible condition, an implantable cardioverter defibrillator therapy as secondary prevention of sudden cardiac death was not indicated in our patient [26]. However, the fact that only 51% had achieved a full recovery in a prospective long-term follow-up study including 84 AN patients [27] requires careful further surveillance of our patient.

Takeaway lessons of this case report:

1. Severe electrolyte disturbance due to refeeding or as a result of severe starvation in patients with AN can result in sudden cardiac death even without a preceding cardiac dysfunction
2. Careful monitoring in early phase of refeeding is of major importance
3. Extracorporeal resuscitation is recommended as bridge to recovery treatment strategy in cardiac arrest and acute heart failure in AN

## References

[1] Patel R., Sardar M. R., Greway A. (2015). Cardiovascular impact of eating disorders in adults: a single center experience and literature review. *Heart Views*.

[2] Neumärker K.‐. J. (1997). Mortality and sudden death in anorexia nervosa. *The International Journal of Eating Disorders*.

[3] Beatriz J., Juregui I., Juregui Lobera I. Cardiovascular complications in eating disorders. *Relevant Topics in Eating Disorders. InTech;*.

[4] Mont L., Castro J., Herreros B. (2003). Reversibility of cardiac abnormalities in adolescents with anorexia nervosa after weight recovery. *Journal of the American Academy of Child and Adolescent Psychiatry*.

[5] Attia E., Walsh B. T. (2009). Behavioral management for anorexia nervosa. *The New England Journal of Medicine*.

[6] Solomon S. M., Kirby D. F. (2016). The refeeding syndrome: a review. *Journal of Parenteral and Enteral Nutrition*.

[7] Kohn M. R., Golden N. H., Shenker I. R. (1998). Cardiac arrest and delirium: presentations of the refeeding syndrome in severely malnourished adolescents with anorexia nervosa. *Journal of Adolescent Health*.

[8] Webb G. J., Smith K., Thursby-Pelham F., Smith T., Stroud M. A., Da Silva A. N. (2011). Complications of emergency refeeding in anorexia nervosa: case series and review. *Acute Medicine.*.

[9] Isner J. M., Roberts W. C., Heymsfield S. B., Yager J. (1985). Anorexia nervosa and sudden death. *Annals of Internal Medicine*.

[10] de Simone G., Scalfi L., Galderisi M. (1994). Cardiac abnormalities in young women with anorexia nervosa. *British Heart Journal*.

[11] Birmingham C. L., Gritzner S. (2007). Heart failure in anorexia nervosa: case report and review of the literature. *Eating and Weight Disorders - Studies on Anorexia, Bulimia and Obesity*.

[12] Davidson A., Anisman P. C., Eshaghpour E. (1992). Heart failure secondary to hypomagnesemia in anorexia nervosa. *Pediatric Cardiology*.

[13] Winston A. P., Jamieson C. P., Madira W., Gatward N. M., Palmer R. L. (2000). Prevalence of thiamin deficiency in anorexia nervosa. *The International Journal of Eating Disorders*.

[14] Fonseca V., Havard C. W. (1985). Electrolyte disturbances and cardiac failure with hypomagnesaemia in anorexia nervosa. *BMJ*.

[15] Piza J., Cespedes R., Troper L., Miller J. H., Berenson G. S. (1971). Myocardial lesions and heart failure in infantile malnutrition. *The American Journal of Tropical Medicine and Hygiene*.

[16] Whitelaw M., Gilbertson H., Lam P. Y., Sawyer S. M. (2010). Does aggressive refeeding in hospitalized adolescents with anorexia nervosa result in increased hypophosphatemia?. *The Journal of Adolescent Health*.

[17] Padfield G. J., Escudero C. A., De Souza A. M. (2016). Characterization of myocardial repolarization reserve in adolescent females with anorexia nervosa. *Circulation*.

[18] Nedvidkova J., Dostalova I., Bartak V., Papezov H., Pacak K. (2004). Increased subcutaneous abdominal tissue norepinephrine levels in patients with anorexia nervosa: an in vivo microdialysis study. *Physiological Research*.

[19] Bonnemeier H., Mall G., Wiegand U. K. H. (2006). Sudden cardiac death due to catecholamine cardiomyopathy in anorexia nervosa. *Resuscitation*.

[20] Templin C., Ghadri J. R., Diekmann J. (2015). Clinical features and outcomes of Takotsubo (stress) cardiomyopathy. *New England Journal of Medicine*.

[21] Santoro F., Núñez Gil I. J., Stiermaier T. (2019). Assessment of the German and Italian Stress Cardiomyopathy score for risk stratification for in-hospital complications in patients with Takotsubo syndrome. *JAMA Cardiology*.

[22] Ono T., Kasaoka S., Fujita M. (2009). Complete recovery from severe myocardial dysfunction in a patient with anorexia nervosa. *Journal of Cardiology*.

[23] Golden N. H., Katzman D. K., Sawyer S. M. (2015). Position paper of the Society for Adolescent Health and Medicine: medical management of restrictive eating disorders in adolescents and young adults. *The Journal of Adolescent Health*.

[24] Mehanna H. M., Moledina J., Travis J. (2008). Refeeding syndrome: what it is, and how to prevent and treat it. *BMJ*.

[25] Soar J., Nolan J. P., Böttiger B. W. (2015). European Resuscitation Council Guidelines for resuscitation 2015: section 3. Adult advanced life support. *Resuscitation*.

[26] Priori S. G., Blomström-Lundqvist C., Mazzanti A. (2015). 2015 ESC guidelines for the management of patients with ventricular arrhythmias and the prevention of sudden cardiac death: the Task Force for the Management of Patients with Ventricular Arrhythmias and the Prevention of Sudden Cardiac Death of the European Society of Cardiology (ESC). Endorsed by: Association for European Paediatric and Congenital Cardiology (AEPC). *European Heart Journal*.

[27] Zipfel S., Lowe B., Reas D. L., Deter H. C., Herzog W. (2000). Long-term prognosis in anorexia nervosa: lessons from a 21-year follow-up study. *The Lancet*.

